# Neumotórax catamenial con fuga aérea persistente

**DOI:** 10.23938/ASSN.1069

**Published:** 2024-03-07

**Authors:** Pablo Andrés Ordóñez Lozano

**Affiliations:** 1 Servicio Aragonés de Salud Hospital Universitario Miguel Servet Servicio de Cirugía Torácica Zaragoza España; 2 Servicio Aragonés de Salud Instituto de Investigación Sanitaria Aragón (IIS Aragón) Zaragoza España

**Keywords:** Neumotórax catamenial, Endometriosis, Pleurodesis, Cirugía Torácica Video-asistida, Catamenial pneumothorax, Endometriosis, Pleurodesis, Thoracic Surgery Video-Assisted

## Abstract

El neumotórax catamenial (NC) es aquel neumotórax espontáneo y recurrente que se presenta en mujeres en edad reproductiva y en relación temporal con la menstruación. Se han descrito múltiples variaciones en cuanto a la relación temporal, aunque suele producirse 24 horas antes del inicio de la menstruación o 72 horas después. Su consideración de patología poco frecuente podría deberse a que sea infradiagnosticada debido a la falta de conocimiento. El diagnóstico de NC no suele ser fácil; depende principalmente de la historia clínica pero también puede ser un diagnóstico quirúrgico o histopatológico. Las estrategias de manejo del NC pueden incluir cualquier combinación de terapia hormonal, pleurodesis, resección de parénquima pulmonar y resección/reparación del diafragma.

Se presenta este caso de neumotórax catamenial de manifestación atípica para resaltar la importancia de tener un adecuado conocimiento de esta enfermedad que, por su aparente baja incidencia, puede pasar desapercibida.

## Abstract (en)

Catamenial pneumothorax is a spontaneous recurrent pneumothorax that occurs in women of reproductive age and in temporal relationship with the menses. It usually occurs within 24 hours before or 72 hours after the start of the menstrual cycle; however, multiple variations have been described in terms of the temporal relationship. To date, it is considered a rare condition; this may be justified by the limited available knowledge on this disease leading to misdiagnosis. Often, making a diagnosis of catamenial pneumothorax is complicated; it mainly depends on the medical history; also, it may be a surgical or histopathological diagnosis. Management strategies for catamenial pneumothorax may include combinations of hormonal therapy, pleurodesis, lung parenchyma resection, and diaphragm resection/repair.

The aim of reporting this atypical case of catamenial pneumothorax is to highlight the relevance of having the adequate knowledge on this entity so it does not go unnoticed despite its apparent low incidence.

## INTRODUCCIÓN

El neumotórax catamenial (NC) se define como aquel neumotórax espontáneo y recurrente que se presenta en mujeres en edad reproductiva y en relación temporal con la menstruación; suele producirse 24 horas antes del inicio de la misma o 72 horas después, aunque se han descrito múltiples variaciones^1^. En su mayoría (más del 80%) se presentan en el lado derecho^1^.

Se ha considerado como una patología poco frecuente, representando entre el 3% y 6% de todos los neumotórax espontáneos^1^^,^^2^. No obstante, esta baja frecuencia puede estar justificada por la falta de conocimiento de esta enfermedad, llegando a ser infradiagnosticada. Por otra parte, el NC es la manifestación clínica más común del síndrome de endometriosis torácica, ocurriendo hasta en el 73% de los casos, siendo más frecuente entre los 30 y 40 años^1^.

El diagnóstico de NC depende principalmente de la historia clínica (relación temporal con la menstruación), mientras que el diagnóstico de neumotórax relacionado con la endometriosis se basa en la inspección visual intraoperatoria y el examen histológico de las lesiones características, las cuales dependen en gran medida del conocimiento de la enfermedad, ya que pueden pasarse por alto fácilmente^1^.

Las estrategias de manejo del NC pueden incluir cualquier combinación de terapia hormonal, pleurodesis, resección de parénquima pulmonar y resección/reparación del diafragma^3^.

Presentamos el caso de una paciente con neumotórax catamenial con fuga aérea persistente que por su manifestación atípica permite resaltar la importancia de tener un adecuado conocimiento de esta enfermedad que, por su aparente baja incidencia, puede pasar desapercibida.

## CASO CLÍNICO

Paciente de 47 años que acudió al Servicio de Urgencias por dolor torácico derecho asociado a disnea de moderados esfuerzos de cinco días de evolución. Antecedentes de asma y endometriosis que recibió tratamiento hormonal; además, antecedente quirúrgico de resección pulmonar sublobar atípica de distrofia bullosa apical del lóbulo superior derecho (LSD) más pleurectomía mediante cirugía torácica video-asistida (VATS) a los 45 años, por neumotórax espontáneo derecho con fuga aérea persistente.

**Figura 1:**
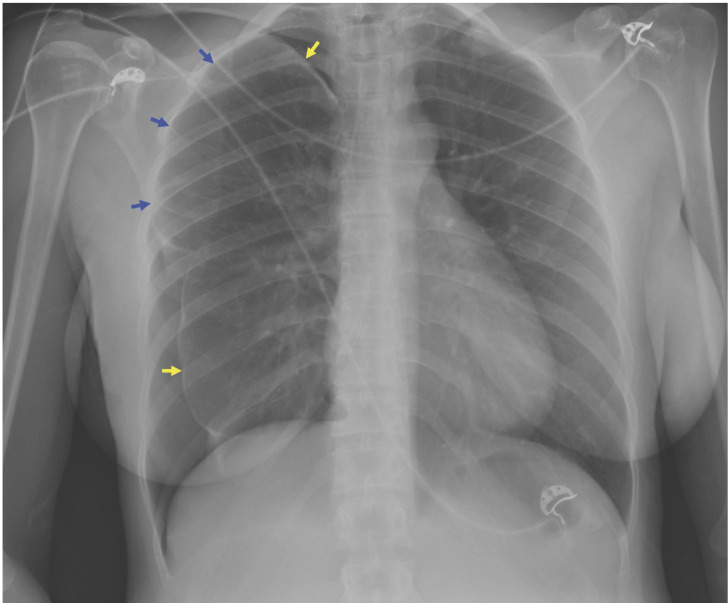
Radiografía de tórax que muestra neumotórax de distribución atípica laterobasal y apical derecho (flechas amarillas) con signos de adherencia del lóbulo superior derecho (flechas azules) en relación con pleurectomía previa.

En la radiografía (Rx) de tórax, se observó neumotórax de distribución atípica parcial laterobasal y apical derecho (Fig. 1) que requirió la colocación de un drenaje torácico como manejo inicial. Se realizó una tomografía axial computarizada (TAC) de tórax para ampliar el estudio, sin observarse alteraciones pulmonares relevantes. Durante el ingreso hospitalario, la paciente refirió que el inicio de los síntomas aparece de manera sincrónica con la menstruación.

**Figura 2:**
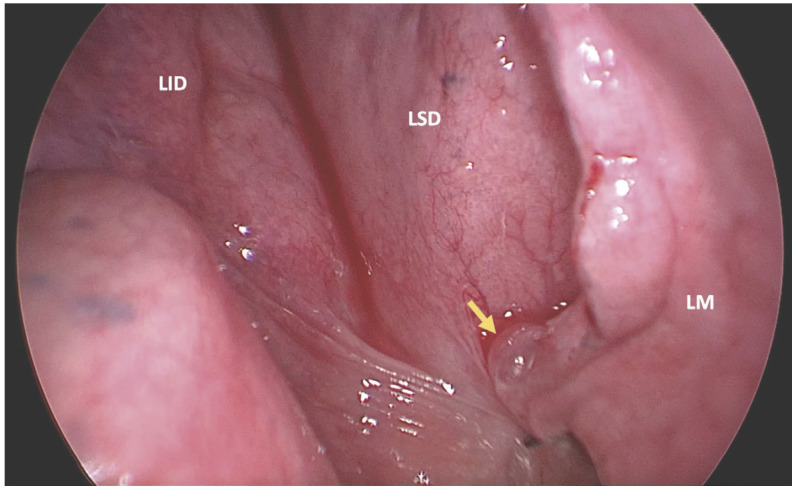
Visión videotoracoscópica intraoperatoria. Se observa *bleb* (flecha amarilla) localizado en la encrucijada cisural. LSD: lóbulo superior derecho. LM: lóbulo medio. LID: lóbulo inferior derecho.

**Figura 3:**
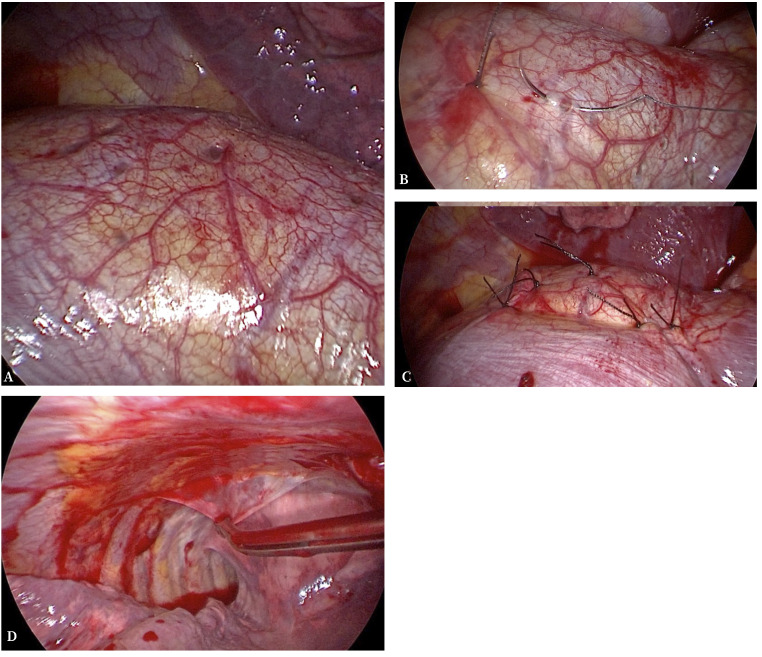
Visión videotoracoscópica intraoperatoria. **A**. Fenestraciones diafragmáticas. **B**. Plicatura y cierre de la fenestración con punto simple de seda 3/0. **C**. Resultado final del cierre de las fenestraciones diafragmáticas. **D**. Pleurectomía.

Dada la recidiva ipsilateral, la presencia de fuga aérea persistente en este segundo episodio y la sospecha diagnóstica de NC, se realizó una intervención quirúrgica programada mediante VATS biportal, bajo anestesia general e intubación bronquial selectiva. Durante la cirugía, se observó una amplia adherencia pleuropulmonar firme del LSD, probablemente relacionada con el antecedente quirúrgico, pequeña zona de fuga aérea a nivel de *bleb* en la encrucijada cisural (Fig. 2) y múltiples fenestraciones milimétricas en la región tendinosa del diafragma. Se procedió al cierre de las pequeñas fenestraciones mediante plicaturas con puntos simples de seda 3/0, se aplicó un sellante en la zona de fuga aérea y, como refuerzo, a nivel del diafragma; además, se realizó pleurectomía del campo medio e inferior del hemitórax derecho (Fig. 3).

La paciente tuvo una adecuada evolución postoperatoria. El drenaje torácico se retiró a las 24 horas de la intervención y recibió el alta hospitalaria a las 48 horas de la cirugía. El informe anatomopatológico de la pleurectomía indicó la presencia de signos compatibles con hiperplasia mesotelial reactiva.

Durante el seguimiento ambulatorio la paciente evoluciona adecuadamente, sin recidiva del neumotórax.

## DISCUSIÓN

El neumotórax, se define como la presencia de aire dentro del espacio pleural, que modifica la presión subatmosférica (negativa) intrapleural y ocasiona colapso pulmonar parcial o total. Este puede clasificarse, según la etiología, en espontáneo y adquirido (iatrogénico y traumático). El espontáneo se divide a su vez en primario (cuando no hay enfermedad pulmonar evidente), secundario (cuando hay enfermedad pleuropulmonar subyacente) y catamenial^4^. Este último se define como aquel neumotórax espontáneo, recurrente, que se manifiesta en mujeres en edad reproductiva y en relación temporal con la menstruación^1^. También, existe el neumotórax no catamenial relacionado con la endometriosis, es decir, neumotórax con endometriosis intratorácica comprobada con o sin defectos diafragmáticos, pero sin relación temporal con la menstruación^1^^,^^2^.

Se han planteado varias teorías sobre su etiopatogenia^1^^,^^5^.

- La *hipótesis fisiológica* por niveles elevados de prostaglandina F2 durante la menstruación que ocasionan vasoconstricción y broncoespasmo induciendo la ruptura alveolar o de bullas preexistentes susceptibles de romperse durante estos cambios hormonales.
- La teoría de *microembolización metastásica o linfovascular* de tejido endometrial hasta los pulmones y posterior necrosis catamenial de los focos parenquimatosos endometriales próximos a la pleura visceral, provocando fuga aérea y neumotórax.
- La teoría del *paso de aire transgenital-transdiafragmático* a través de defectos diafragmáticos congénitos o adquiridos (secundario a endometriosis).
- La teoría del *paso de aire transgenital-transdiafragmático* a través de defectos diafragmáticos congénitos o adquiridos (secundario a endometriosis).
- La teoría de la *migración* por menstruación retrógrada que causa la *siembra* pélvica de tejido endometrial y su posterior migración hacia áreas subdiafragmáticas. Después se produce la necrosis catamenial de estos implantes endometriales diafragmáticos, originando las perforaciones diafragmáticas por las que pasará el tejido endometrial, diseminándose hacia la cavidad torácica.

Los síntomas descritos más frecuentes son dolor torácico, disnea y tos^1^. El diagnóstico de NC no suele ser fácil, depende principalmente de la historia clínica (por la relación temporal con la menstruación), puede ser un diagnóstico quirúrgico mediante una cuidadosa inspección visual intraoperatoria (ausencia de signos de endometriosis en el pulmón o en el diafragma, pero hay hallazgos sospechosos de NC como los defectos diafragmáticos) o histológico-patológico de las lesiones características (signos de endometriosis diagnosticada histológicamente en las muestras resecadas). Por todo ello, es necesario un alto nivel de sospecha y conocimiento de la enfermedad para su diagnóstico ya que puede pasarse por alto fácilmente^1^^,^^6^. En este caso, el primer episodio se consideró como un neumotórax espontáneo (primario) con fuga aérea persistente y tras la segunda cirugía, por los hallazgos intraoperatorios y la clínica referida, se diagnosticó como neumotórax catamenial.

En cuanto a los defectos diafragmáticos (descritos como perforaciones, fenestraciones, agujeros o poros), estos pueden ser únicos o múltiples, diminutos de unos cuantos milímetros -como en nuestro caso- hasta grandes defectos mayores de un centímetro; incluso se han descrito hernias hepáticas parciales asociadas^7^. Generalmente, estos defectos suelen estar localizados en la región tendinosa central, a menudo adyacentes a nódulos coexistentes. La presencia de tejido endometrial, que se encuentra ocasionalmente en los bordes de los defectos, respalda la teoría de que estos representan la ruptura cíclica por una propiedad erosiva de los implantes endometriales^1^^,^^8^.

En el tratamiento del NC, la colocación del drenaje torácico constituye el primer procedimiento a realizar en la mayoría de los casos. El manejo quirúrgico mediante VATS es el de elección. En ocasiones, puede ser necesario realizar un abordaje abierto por toracotomía cuando se requiere una reparación extensa del diafragma, o en casos de reintervenciones^1^. En este caso, a pesar de considerarse una reintervención ipsilateral, los hallazgos radiológicos (adherencia pleuropulmonar localizada solo en el LSD sin otras alteraciones pulmonares relevantes) e intraoperatorios (defectos diafragmáticos milimétricos), permitieron realizar el abordaje mediante VATS.

En general, durante la cirugía es importante realizar una adecuada inspección identificando bullas o *blebs*, localizar las zonas de fuga aérea, explorar el diafragma en búsqueda de defectos, manchas o nódulos, así como la inspección de la pleura parietal, el pulmón y el pericardio. Asimismo, es preciso realizar la pleurodesis o pleurectomía parcial.

Específicamente, para abordar la patología diafragmática se han descrito plicaturas y/o resección del área diafragmática afectada, así como coberturas del diafragma con mallas de poliglactina, polipropileno, politetrafluoroetileno (PTFE) o parche de pericardio bovino, con buenos resultados a mediano plazo^1^^,^^3^. En nuestro caso, la paciente presentaba una fuga aérea a nivel de *bleb* de localización intercisural y profunda que dificultaba el abordaje para realizar la resección de la misma. Se observaron varias y pequeñas fenestraciones diafragmáticas que se cerraron mediante sutura manual y se completó la pleurectomía del campo medio e inferior.

Hay estudios que demuestran una mayor tasa de recidiva tras únicamente terapia hormonal en comparación con la resección/reparación diafragmática. Las tasas de recidiva más bajas se lograron cuando se utilizó una combinación de intervención quirúrgica que incluyera la reparación diafragmática, pleurodesis y terapia hormonal^3^. Esta última se suele indicar como complemento de la cirugía con la intención de prevenir las recidivas del neumotórax catamenial y/o relacionado con la endometriosis, mediante la supresión de la actividad del endometrio ectópico, hasta lograr formar adherencias pleurales efectivas^1^^,^^9^.

En conclusión, ante la presencia de un neumotórax espontáneo recidivante en mujeres en edad reproductiva siempre hay que tener presente la posibilidad de encontrarnos ante un neumotórax catamenial o neumotórax no catamenial relacionado con la endometriosis. En primer lugar, es necesario valorar la posible relación temporal con la menstruación y, en caso de indicarse cirugía, realizar una adecuada inspección sistemática del pulmón, el diafragma, la pleura parietal y el pericardio en busca de lesiones características.
